# Helicopter emergency medical service dispatch in older trauma: time to reconsider the trigger?

**DOI:** 10.1186/s13049-021-00877-3

**Published:** 2021-05-07

**Authors:** J. E. Griggs, J. W. Barrett, E. ter Avest, R. de Coverly, M. Nelson, J. Williams, R. M. Lyon, on behalf of Air Ambulance Kent Surrey Sussex

**Affiliations:** 1 Air Ambulance Kent Surrey Sussex, Hanger 10 Redhill Aerodrome, Redhill, Surrey, RH1 5YP UK; 2 University of Surrey, Guilford, GU2 7XH UK; 3 South East Coast Ambulance Service NHS Foundation Trust, Nexus House, 4 Gatwick Road, Crawley, RH10 9BG UK; 4 Department of Emergency Medicine, University Medical Center Groningen, Groningen, The Netherlands; 5 Royal Sussex County Hospital, Eastern Road, Brighton, BN2 5BE UK; 6 University of Hertfordshire, College Lane, Hatfield, Hertfordshire, AL10 9AB UK

**Keywords:** Older trauma, Dispatch sensitivity, Critical care interventions, Helicopter emergency medical service

## Abstract

**Background:**

Helicopter Emergency Medical Services (HEMS) respond to serious trauma and medical emergencies. Geographical disparity and the regionalisation of trauma systems can complicate accurate HEMS dispatch. We sought to evaluate HEMS dispatch sensitivity in older trauma patients by analysing critical care interventions and conveyance in a well-established trauma system.

**Methods:**

All trauma patients aged ≥65 years that were attended by the Air Ambulance Kent Surrey Sussex over a 6-year period from 1 July 2013 to 30 June 2019 were included. Patient characteristics, critical care interventions and hospital disposition were stratified by dispatch type (immediate, interrogate and crew request).

**Results:**

1321 trauma patients aged ≥65 were included. Median age was 75 years [IQR 69–89]. HEMS dispatch was by immediate (32.0%), interrogation (43.5%) and at the request of ambulance clinicians (24.5%). Older age was associated with a longer dispatch interval and was significantly longer in the crew request category (37 min [34–39]) compared to immediate dispatch (6 min [5–6] (*p* = .001). Dispatch by crew request was common in patients with falls < 2 m, whereas pedestrian road traffic collisions and falls > 2 m more often resulted in immediate dispatch (*p* = .001). Immediate dispatch to isolated head injured patients often resulted in pre-hospital emergency anaesthesia (PHEA) (39%). However, over a third of head injured patients attended after dispatch by crew request received PHEA (36%) and a large proportion were triaged to major trauma centres (69%).

**Conclusions:**

Many patients who do not fulfil the criteria for immediate HEMS dispatch need advanced clinical interventions and subsequent tertiary level care at a major trauma centre. Further studies should evaluate if HEMS activation criteria, nuanced by age-dependant triggers for mechanism and physiological parameters, optimise dispatch sensitivity and HEMS utilisation.

**Supplementary Information:**

The online version contains supplementary material available at 10.1186/s13049-021-00877-3.

## Introduction

Advances in healthcare have enabled greater independence and activity in older people [1, 2]. This has led to a greater prevalence of older trauma, with 50% of severely injured patients over the age of 65-years recorded on the Trauma Audit and Research Network (TARN) [2, 3]. Inevitably, growing demand is being placed on acute healthcare services. The outcome of older trauma patients is difficult to predict; however, published studies indicate an increased risk of morbidity and mortality, with a mortality rate of 50% in adults aged over 75 years [4].

Helicopter Emergency Medical Services (HEMS) provide enhanced pre-hospital medical care to major trauma victims. HEMS can deliver specialist interventions such as pre-hospital hospital emergency anaesthesia (PHEA). Accurate tasking of HEMS is important to deliver this valuable resource. Accurate HEMS dispatch is critical to the appropriate activation of an enhanced care team to those patients whom may benefit most from advanced critical care interventions [5–7]. Various dispatch algorithms enhance sensitivity by coupling mechanism with anatomical and physiological criteria [7]. These have been shown to decrease HEMS activations to 55% of trauma patients, whilst at the same time accurately directing the enhanced care team to higher acuity patients [8].

Emergency medical dispatchers track an established pathway during a 112/999 call to discern traumatic injuries, of which the dispatch triggers are largely validated in the adult trauma population [9] and not specifically adapted to the older trauma subgroup. Clinical observations of traumatically injured older adults may present within parameters equivalent to younger ‘well’ adults [3]. Therefore, injury severity is potentially masked to both the caller and clinician, and subsequently HEMS dispatch is delayed [10].

In the present study, we sought to explore HEMS dispatch accuracy in older trauma patients by analysing HEMS specific interventions and disposition of this subgroup of trauma patients, stratified by dispatch type.

## Methods

### Study design and setting

We performed a retrospective cohort study of HEMS dispatch to older trauma in the south-east of England between 1 July 2013 to 30 June 2019.

Air Ambulance Kent Surrey Sussex (AAKSS) serves a population of approximately 4.3 million, with a transient population of up-to 11 million. AAKSS attends approximately 1600 patients each year. Two doctor-paramedic teams deploy by helicopter or response car, one of which operates a 24-h day and the other an 18-h day. The HEMS team brings advanced clinical procedures to complement the scope of practice provided by a land based Critical Care Paramedic (CCP), to include: pre-hospital emergency anaesthesia (PHEA), advanced analgesia and sedation, blood product transfusion and surgical intervention (thoracostomy and thoracotomy). The service works alongside the regional ambulance service of South East Coast Ambulance Service (SECAmb).

A CCP and HEMS dispatcher evaluate and task the critical care resources across the region from the Emergency Operations Centre (EOC). The tasking algorithm was devised internally and is previously published [11]. Activations are categorised as: immediate, interrogated or crew request. Immediate dispatch is triggered by pre-determined criteria. Interrogated dispatch is triggered where subsequent clinical information is reviewed, and HEMS dispatch agreed. Both immediate and interrogate dispatches are based on mechanism of injury (MOI), clinical condition of the patient and geographical location. A crew request can be activated by crews on scene (figure 3, suppl. file).

### Study population

All older trauma patients (≥65 years) attended by HEMS with suspected traumatic injuries during the study period were included. Exclusion criteria comprised: patients < 65 years and patients presenting with suspected medical aetiology. Inter-facility transfers were excluded due to the unknown interventions delivered prior to HEMS arrival, and mutual aid requests excluded due to a variable, and unaccounted passage of time prior to AAKSS receiving the tasking.

### Data collection

An electronic record system (HEMSbase Medic One Systems, Ltd. UK) is used at AAKSS. The following data were retrieved from the electronic patient record: patient identification number, timings (112/999-time, dispatch interval). Patient characteristics (age, gender), mechanism of injury (assault [blunt/penetrating], fall (< 2 m [m], > 2 m), intentional self-harm, road traffic collisions and other (for example, crush injury)), anatomical site of injury (head, neck, thorax, abdomen, upper leg, upper arm), GCS, advanced pre-hospital interventions provided by the HEMS team, drugs administered, patient disposition (pronounced life extinct on scene, or transport to local hospital, Trauma Unit or MTC), and conveyance (carry, ground escort, or ground assist) by transport modality were retrieved.

Advanced pre-hospital interventions comprise those not performed by ground ambulance teams where a CCP is not present: pre-hospital emergency anaesthetic (PHEA), open finger thoracostomy, resuscitative thoracotomy, ultrasound sonography (USS), administration of prothrombin complex concentrate (PCC, beriplex®), insertion of intercostal chest drain (ICD), administration of intravenous (IV) antibiotics and/or antiviral drugs, administration of hypertonic saline 5%, advanced analgesia (fentanyl and ketamine), and pre-hospital transfusion therapy. Transfusion therapy consisted of packed red blood cells (PRBC) and freeze-dried plasma (FDP). PRBCs were available throughout the study period and FDP (*Lyoplas*) available from 3 April 2015.

Data extraction and eligibility of patients was performed by one of the authors (JG) and any inaccuracies and discrepancies were resolved by a second author (JB). These included miscategorisation of calls with regard to mechanism, and coding of variables.

### Ethical considerations

This project met National Institute for Healthcare Research (NIHR, UK) criteria for service evaluation and formal ethical approval was therefore not required. The project was approved by the Research & Development Committee at AAKSS.

### Statistical analysis

Descriptive statistics are given as mean [95% CI] or median [IQR]. Patients were stratified into three groups according to age: 65–74, 75–84, 85 and over. Comparisons across groups were made using Chi-square or Kruskal-Wallis tests where appropriate. Where statistical significance was found (set at a *p*-value < 0.05) Dunn’s post-hoc testing with Bonferroni correction was performed. The study applied Strengthening the Reporting of Observational Studies in Epidemiology (STROBE) Guidelines [12], with missing values reported. All statistical analyses were conducted using SPSS 26.0 (IBM).

## Results

**Fig. 1:**
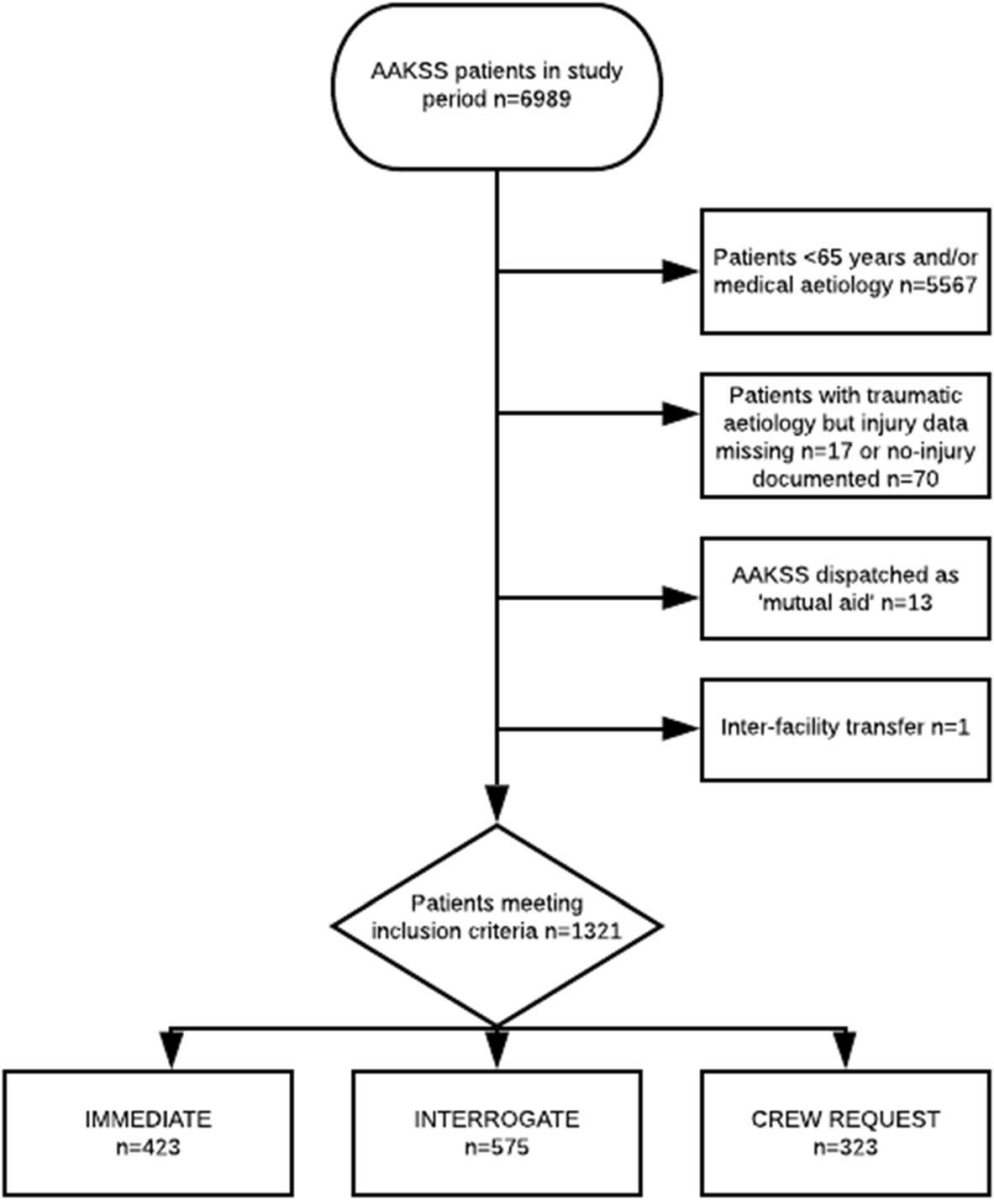
Flow chart of study population

A total of 6989 patients were attended by AAKSS during the study period (Fig. 1). Of these, 1422 were traumatically injured and aged ≥65. Seventy were excluded because “no injury” was recorded and the injury was of presumed medical aetiology. Seventeen cases were removed due to missing injury data, 13 as they were “mutual aid” requests and 1 as it was an inter-facility transfer. The resulting study population included 1321 patients ≥65 years, with 423 (32.0%) immediate dispatches; 575 (43.5%) interrogated dispatches; and 323 (24.5%) crew requests. During the study period (for all patients), the distribution by dispatch type was; immediate dispatch formed the greatest proportion (40%), followed by interrogate (24%) and crew requests (30%).

### Study population characteristics

Males represented 62% (*n* = 832) of the study population with a median age of 75 years (IQR 69–81). When stratified by age-group, 65–74 year olds represented 49% (*n* = 675) of the patients attended, 75–84 year olds represented 35% of patients (*n* = 468), and 14.8% (*n* = 196) were over 85 years As expected, the dispatch interval from 112/999 call to arrival of the HEMS team on scene was longer for crew requests (37 min [34–39]) compared to immediate (6 min [5–6]) or interrogated dispatch (15 min [13–16], *p* < .001) (Table 1). This was representative of dispatch for the whole cohort of patients attended by AAKSS during the study period for immediate dispatch (7 min [6–9]), interrogated dispatch (9 min [6–12]) and crew request (36 min [30–41]).

**Table 1:** Study population demographics, mechanism, presentation, anatomical injury site and patient disposition

	Total	Immediate	Interrogate	Crew request	*P value*
**Demographics**	n [%]	n [%]	n [%]	n [%]	
Patients	1321 [100]	423 [32.0]	575 [43.5]	323 [24.5]	.001^1^
Age, years (n, IQR)	75 [69–81]	73 [68–80]	75 [69–81]	76 [70–82]	.608^2^
65–74	657 [49.7]	226 [53.4]	280 [48.7]	151 [46.8]	.157^1^
75–84	468 [35.4]	136 [32.1]	218 [37.9]	114 [35.3]	.170^1^
> 85	196 [14.8]	61 [14.4]	77 [13.4]	58 [17.7]	.174^1^
Males	832 [62.9]	289 [68.3]	354 [61.6]	189 [58.5]	.15^1^
999-dispatch time (mins, mean - 95%CI)	18 [16.5–18.9]	6.3 [5.7–6.9]	15.1 [13.4–16.8]	37.1 [34.4–39.9]	.001^3^
**Mechanism**	1321 [100]	423 [32.0]	575 [43.5]	323 [24.5]	.001
Assault [blunt]	1 [100]	0 [0]	0 [0]	1 [1.0]	.213
Assault [penetrating]	13 [100]	6 [46.1]	3 [23.0]	4 [30.7]	.213
Fall < 2 m	120 [100]	15 [12.5]	43 [35.8]	62 [51.6]	.001
Fall > 2 m	388 [100]	132 [34.0]	144 [37.1]	112 [28.8]	.006
ISH other	10 [100]	4 [40.0]	4 [40.0]	2 [20.0]	.856
ISH sharp	24 [100]	10 [41.6]	14 [58.3]	0 [0]	.005
Other	103 [100]	23 [22.3]	47 [45.6]	33 [32.0]	.49
RTC cyclist	66 [100]	24 [36.3]	28 [42.4]	14 [21.2]	.712
RTC driver	246 [100]	73 [29.6]	130 [52.8]	43 [17.4]	.002
RTC passenger	103 [100]	35 [33.9]	51 [49.5]	17 [16.5]	.14
RTC pedestrian	212 [100]	87 [41.0]	98 [46.2]	27 [12.7]	.001
RTC motorcyclist	35 [100]	14 [40.0]	13 [37.1]	8 [22.8]	.58
**Presentation**					
TCA	99 [7.4]	71 [16.8]	14 [2.4]	14 [4.3]	.001
Isolated trauma	622 [47.0]	192 [45.4]	266 [46.2]	164 [50.8]	.304
Polytrauma	633 [47.9]	217 [51.3]	266 [46.2]	150 [46.4]	.242
**Anatomical injury site**	4743 [100]	1510 [31.84]	43.28 [43.28]	1180 [24.88]	.001
Head	897 [67.9]	307 [34.2]	350 [39.0]	240 [26.7]	.001
Neck	162 [12.3]	47 [29.0]	74 [45.6]	41 [25.3]	.683
Thorax	544 [41.1]	193 [35.4]	229 [42.0]	122 [22.4]	.066
Abdomen	301 [22.8]	109 [36.2]	128 [42.5]	64 [21.2]	.145
Upper arm	172 [13.0]	54 [31.3]	77 [44.7]	41 [23.8]	.95
Upper leg	143 [10.8]	36 [25.1]	70 [48.9]	37 [25.8]	.168
**Transported patients**	1204 [19.1]	341 [80.6]	550 [95.7]	313 [96.9]	.001
Carry	407 [30.8]	125 [29.5]	163 [28.4]	119 [36.8]	.022
Escort	369 [27.9]	109 [25.7]	147 [25.6]	113 [34.9]	.005
Assist	438 [33.1]	112 [26.44]	243 [42.3]	83 [25.7]	.001
PLE	106 [8.0]	77 [18.2]	22 [3.9]	7 [2.2]	.001
Unknown	1 [0.1]	0 [0]	0 [0]	1 [0.3]	.245
**Destination**					
MTC	793 [60.0]	244 [57.7]	324 [56.4]	225 [69.7]	.001
TU	374 [28.3]	91 [21.5]	202 [35.1]	81 [25.1]	.001
LEH	37 [2.8]	6 [1.4]	24 [4.1]	7 [2.2]	.024
Unknown	11 [0.8]	5 [1.2]	3 [0.5]	3 [1.0]	.549

Values are given as frequency (n) and percentage (%). ISH Intentional self-harm, RTC Road traffic collision, TCA Traumatic cardiac arrest, PLE Pronounced life extinct, MTC Major trauma centre, TU Trauma unit, LEH Local emergency hospital

**Fig. 2:**
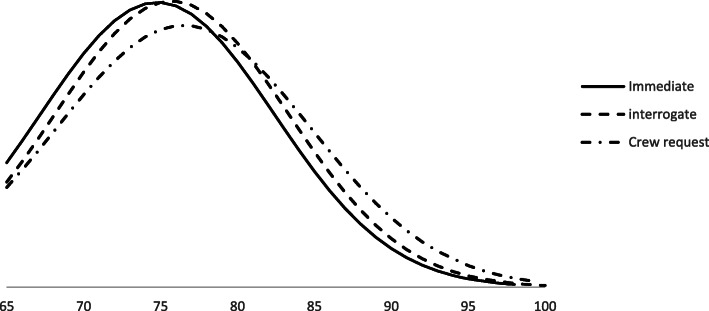
The distribution of the older trauma cohort when age is plotted against dispatch criteria

Figure 2 shows that the median age for all three dispatch types was 75 (IQR 69–81). Median patient age increased as dispatch priority decreased (Table 1).

### Mechanism, anatomical injury site and transport modality in relation to dispatch type

When HEMS were dispatched, falls from height (> 2 m) and RTCs were the most frequent mechanism encountered. Overall, frequency distribution of mechanisms for the three dispatch groups were significantly different (*p* = .001). Direct dispatches were significantly more prevalent in falls > 2 m (*p* = .006) and when pedestrians were involved in road traffic collisions (RTCs) (*p* < .001), whereas drivers involved in an RTC were more likely to trigger an interrogated dispatch (*p* = .001). Falls < 2 m were likely to trigger a dispatch on crew request (*p* = .001) (Table 1).

Overall, head injuries were the most frequently reported injury (*n* = 897, 67%) followed by injuries to the thorax (*n* = 544, 41%, Table 1). Overall there was no significant difference in the frequency distribution of anatomical injury sites (*p* = .162), however, between dispatch groups, a significant difference in head injuries was observed (*p* = .001). Post hoc analysis indicated that immediate and crew requests were significantly more prevalent than interrogate (*p* = .012 and *p* = .004, respectively). There was no significant difference in the frequency distribution of poly and isolated trauma (*p* = .358). The majority of patients were transported to hospital (*n* = 1204, 91%) of which 60% conveyed directly to an MTC (*n* = 793). The proportion of patients transported to an MTC (69%) was higher after a crew request dispatch than after an immediate or interrogate dispatch (*p* = .001).

### Advanced interventions

Table 2 displays the advanced interventions performed during the study period. PHEA was performed in a greater proportion (*n* = 86, 26%) of patients attended by crew request compared to immediate dispatch (*p* = .001) and interrogate dispatch (*p* = .001). PRBCs and FDP were more often administered in the immediate dispatch group (13 and 12%, respectively) compared to the interrogate group 5% (*p* = .004) and 4% (*p* = .004), and the crew request group 3% (*p* = .002) and 4.3% (*p* = .032). Advanced analgesia in the form of ketamine was administered similarly across all groups (*p* = .777), as was hypertonic saline (*p* = .111).

**Table 2:** Advanced interventions performed in the patient cohort stratified by dispatch

	Total	Immediate	Interrogate	Crew Request	*P value*
	n [%]	n [%]	n [%]	n [%]	
Patients	1321 [100]	423 [32.0]	575 [43.5]	323 [24.5]	
PHEA	260 [19.7]	93 [22.0]	81 [14.1]	86 [26.6]	.001
PRBC	97 [7.3]	55 [13]	30 [5.2]	12 [3.7]	.001
FDP	92 [7.0]	54 [12.8]	24 [4.1]	14 [4.3]	.001
Thoracotomy	4 [0.3]	2 [0.5]	2 [0.4]	0 [0]	.334*
Thoracostomy	178 [13.44]	119 [28.1]	39 [6.7]	20 [6.1]	.001
Antibiotic therapy	86 [6.5]	20 [44.7]	46 [8]	20 [6.1]	.153
USS	490 [37.0]	183 [43.3]	200 [34.8]	107 [33.1]	.009
Anticoagulant reversal	28 [2.1]	10 [2.3]	7 [1.2]	11 [3.4]	.083
Fentanyl	114 [8.6]	38 [8.9]	53 [9.2]	23 [7.1]	.535
Ketamine	127 [9.6]	38 [8.9]	55 [9.6]	34 [10.5]	.777
Morphine	238 [18.0]	55 [13]	109 [18.9]	74 [22.9]	.002
Hypertonic saline 5%	76 [5.7]	23 [5.4]	27 [4.7]	26 [8.0]	.111
Chest drain	7 [0.5]	1 [0.2]	3 [0.5]	3 [0.9]	.77*

*PHEA* Prehospital emergency anaesthesia, *PRBC* Packed red blood cells, *FDP* Freeze dried plasma, *USS* Ultrasound sonography. *Fisher-exact test

### Isolated head injury

Over a third of all older trauma patients that HEMS attended had a documented isolated head injury (*n* = 410) and a third of these received PHEA (*n* = 114, 27%), despite that median presenting GCS of these patients was 14. Patients most often received PHEA following a crew request (35%) especially when the presenting GCS was < 8 (*p* = .005). Anticoagulant reversal was administered in a similar proportion of patients across each dispatch type (*p* = .152) (Table 3).

**Table 3:** Isolated head injury stratified by dispatch criteria

	Total (*n* = 410)	Immediate	Interrogate	Crew Request	*P value*
	n [%]	n [%]	n [%]	n [%]	
Age (median, IQR)	75 [13]	74 [13.5]	75 [13]	77 [13.5]	.019
GCS (median, IQR)	14 [6.8]	13 [7]	14 [5]	13 [7.5]	.45
Missing GCS	26 [6.3]	14 [10.5]	8 [5.1]	4 [3.3]	.045
PHEA	114 [27.9]	38 [28.6]	33 [21.2]	43 [35.5]	.029
Anticoagulant reversal	11 [2.7]	3 [2.3]	2 [1.2]	6 [4.9]	.152
Hypertonic saline 5%	35 [8.6]	12 [9.0]	11 [7.1]	12 [9.9]	.716
GCS ≤8	96 [23.4]	34 [25.6]	29 [18.6]	33 [27.3]	.327
GCS ≤8 with PHEA	68 [16.6]	19 [55.9]	19 [65.6]	30 [91.0]	.005

Isolated head injury stratified by dispatch criteria. *GCS* Glasgow coma scale, *PHEA* Pre-hospital emergency anaesthesia

## Discussion

In this retrospective cohort analysis on HEMS dispatch to older trauma, we demonstrate that many older trauma patients who do not fulfil the initial criteria for immediate dispatch, need advanced interventions and subsequent tertiary level care at a major trauma centre.

Older trauma represents 20% of all major trauma in the UK [4] and projections suggest that by 2040 one in four people will be aged 65 or over [3]. With the greatest proportion attended aged between the ages of 65–74 years, older trauma represents a significant proportion (20%) of all trauma attended by HEMS, consistent with the current literature [1, 2, 13]. The increasing national trend may be attributed to improved detection and documentation [14], however, the explanation for increasing older trauma seen by HEMS is more likely due to the proportional increase of older people and greater independence, and comprehensive clinical assessment by on-scene clinicians.

HEMS are often associated with major trauma as a result of significant mechanism; however, we report that in an older patient, HEMS are frequently requested after an innocuous mechanism. Falls from < 2 m comprised a significant proportion of the patients attended in our study, which is consistent with both wider literature [3, 15–17] and TARN data where falls from standing height contribute significantly to the mechanism of injury [2]. Low impact trauma and specifically low energy falls result in 30% of serious injury in patients > 65 years, compared to 4% in < 65 years; and are 10 times more likely to cause death [15, 18]. Only 33–50% of older trauma patients protect themselves with outstretched arms, compared to 90% of younger adults [4]. This exposes patients to head, neck and thoracic injuries with a high injury severity that is disproportionate to mechanism [14], with associated worse functional outcomes regardless of injury severity [19, 20]. With a heavier weighting on mechanism in our dispatch criteria we could argue it is not suitably adapted to the older trauma patient, hence there are a greater proportion of crew requests than in patients < 65 years.

In our study, the likelihood that HEMS was dispatched on request of a ground crew instead of immediately or after interrogation occurred with a higher frequency with increasing age. As previously noted, serious injury can be masked and clinicians at scene have limited diagnostic tools to aid them in injury identification [18]. The implication of undetected occult injuries, insensitivity of triage tools and clinical decision-making in pre-hospital care is under-triage [18, 21–23]. Under-triage is reported to be as high as 58% in patients aged 90 and over [24]. Thus, further exploratory analysis is required on the characterisation of older trauma patients whom are under or over-triaged within trauma networks to feedback into the decision-making of initial clinicians.

Advanced pre-hospital critical care interventions were performed in a high proportion of older trauma activations. We report that even when HEMS was delayed to dispatch, and a passage of time exists whilst the ground ambulance crew assess the patient, a high proportion still required advanced interventions. One such intervention is PHEA, which was increasingly performed when the dispatch category was crew request, irrespective of whether the patient had polytrauma or isolated head injury. It is well documented that older adults with a significant head injury present with a higher functioning GCS compared to younger adults with the same severity of injury, and that GCS is not a good representation of injury severity [25, 26]. This may contribute to under-triage of these patients during the primary dispatch process.

Advanced interventions provided by HEMS are an unparalleled resource and lowering dispatch triggers without robust data would compromise operational models. Dispatch sensitivity may be enhanced by adapting the algorithm to include mechanism adapted to the older trauma patient. For example, we could consider prioritising a 999/112 call advising us of an older person with potential for traumatic injury in combination with a more moderate mechanism. For example, a pedestrian RTC at a speed of >30mph as opposed to 40mph. Novel approaches which review call communication and conversation analysis between the caller or bystander and call taker may allow us to identify unknown criteria missing from reviews of dispatch accuracy. Similarly, live video-transmission from scene may help gauge physiological parameters and expedite dispatch [27].

A marked proportion of older trauma from the crew request dispatch were conveyed to an MTC with the HEMS team by aircraft or land. The regionalisation of trauma services within the study area necessitates targeted critical care resources, and the time-saving nature of expedited transport by helicopter to definitive care itself is advantageous. In combination both HEMS attendance and transport type has shown a significant benefit from ‘low level falls’ in patients with ISS 9 to 15 [7, 28]. The transportation platform, on-scene management and preferred admission to an MTC alone were deemed contributory to survival benefit [29]. A major trauma pre-alert more commonly results in direct transfer to CT in older patients, which conveys earlier detection of injuries [7]. On a HEMS pre-alert a full trauma team will be assembled, this may not be the case when conveyed by a land crew.

## Limitations

Inherent limitations are common to our retrospective design. First, we had to rely on the data as provided by the HEMS teams. Although there were some missing data, overall data completeness was good due to the use of our electronic patient record with dedicated data entry fields for all patients. Further, no causal relations could be established due to the retrospective design. Finally, our findings cannot be generalized to non-HEMS dispatch, as we appreciate that HEMS only attends a fraction of elderly patients and confounding by indication has most certainly contributed to the relatively high number of HEMS interventions in our study population. Although considered highly relevant in our study cohort, confounding variables such as co-morbidities, polypharmacy and pre-injury status are not reported. Although it could be inferred that HEMS are tasked to the most critical older trauma patients our study lacks sufficient physiology data and patient follow-up to infer the implication of dispatch accuracy on patient benefit.

## Conclusion

Older trauma patients sustain minor injuries warranting HEMS attendance by seemingly innocuous mechanisms, and do not fulfil criteria for immediate HEMS dispatch. However, a high proportion of patients require advanced clinical interventions, such as PHEA, and subsequent tertiary level care at a major trauma centre. Dispatch accuracy may be improved by the addition of physiological parameters to mechanism information. Further studies should evaluate if HEMS activation criteria, nuanced by age-dependant triggers for mechanism and physiological parameters, optimise dispatch sensitivity and HEMS utilisation.

## Supplementary Information

**Additional file 1.** HEMS tasking criteria AAKSS version 2.

## Data Availability

The datasets used and/or analysed during the current study are available from the corresponding author on reasonable request.

## Abbreviations

HEMS: Helicopter Emergency Medical Services
IQR: Interquartile Range
RTC: Road Traffic Collision
PHEA: Pre-hospital Emergency Anaesthesia
MTC: Major Trauma Centre
TARN: Trauma Audit and Research Network
CCP: Critical Care Paramedic
EOC: Emergency Operations Centre
MOI: Mechanism of Injury
PCC: Prothrombin Complex Concentrate
USS: Ultrasound sonography
ICD: Intercostal chest drain
IV: Intravenous
PRBC: Packed Red Blood Cells
FDP: Freeze-dried plasma
NIHR: National Institute for Healthcare Research
CI: Confidence Interval
STROBE: Strengthening the Reporting of Observational Studies in Epidemiology Guidelines
GCS: Glasgow Coma Score
CT: Computed Tomography
