# Supplementary material for: Helicopter emergency medical service dispatch in older trauma: time to reconsider the trigger?
Source: Scand J Trauma Resusc Emerg Med. 2021 May 7;29:62. doi: 10.1186/s13049-021-00877-3 (PMC8103626; doi:10.1186/s13049-021-00877-3)
Supplement: Supplementary file 1 [file 13049_2021_877_MOESM1_ESM.docx]

Supplementary file 1 HEMS tasking criteria AAKSS version 2

HEMS/MERIT Grade 1 rapid dispatch criteria (single trigger)

MECHANISM

M11. Fall from second floor or higher (>20 feet or 6 meters) in an adult

M12. Fall from first floor or higher (>12 feet or 3.5 meters) in children, elderly or frail

M13. Traumatic amputation, de-gloved, impaled or mangled limb (above the wrist or ankle)

M14. RTC person ejected from vehicle compartment

M15. RTC/trauma with person trapped or crushed under or between the vehicle or object

M16. RTC with an associated fatality in the same vehicle compartment

Z1. Mass casualty or major incident

CONDITION

C11. Confirmed traumatic cardiac arrest without obvious injuries incompatible with life

C12. Major blunt or penetrating trauma with indication of a persistent decreased level of consciousness or collapse

C13. Request from other agency (FRS, Police, HM Coastguard)

LOCATION

L11. Sudden or witnessed cardiac arrest if remote or rural for rapid delivery of ALS

L12. Potential major or moderate trauma, or serious medical emergency on farmland, open parkland, downland or a clearly rural, remote or inaccessible location

HEMS/MERIT Grade 2 rapid dispatch criteria (two trigger)

MECHANISM

M21. RTC/trauma with prolonged mechanical entrapment

M22. RTC/trauma - high mechanism including (but not limited to) motorcyclist, cyclist, pedestrian, rollover and equestrian

M23. Major burn, involving face, neck, chest, circumferential or special areas

M24. Serious electrocution injury

M25. Industrial or agricultural incidents or entrapments

M26. Other transportation incidents (rail, air, marine) with confirmed patient

CONDITION

C21. Head trauma with indication of persistent agitation

C22. Blunt trauma with indication of shock or collapse

C23. High impact blunt or penetrating trauma to the chest with shortness of breath, difficultly in breathing or collapse

C24. Blunt trauma with multiple fractures reported, especially injuries both above and below the diaphragm

C25. Complex cardiac arrest or peri-arrest cases including drowning, hypothermia, paediatric, complex illness or complex patient

LOCATION

L21. Mechanism or Condition trigger on farmland, open parkland, downland or a clearly rural, remote or inaccessible location

L22. Mechanism or Condition trigger (excluding complex cardiac arrest) that is greater than 20 minutes from the nearest HEMS resource

L23. Mechanism or Condition trigger with an initial EMS response of >20 minutes

L24. Candidate major trauma patients who are greater than 60 minutes from the nearest MTC
